# RNA-Seq and Iso-Seq Reveal the Important Role of *COMT* and *CCoAOMT* Genes in Accumulation of Scopoletin in Noni (*Morinda citrifolia*)

**DOI:** 10.3390/genes13111993

**Published:** 2022-10-31

**Authors:** Dandan Jia, Can Jin, Shusen Gong, Xuan Wang, Tian Wu

**Affiliations:** Southwest Landscape Architecture Engineering Research Center of State Forestry Administration, Landscape Architecture and Horticulture Science School, Southwest Forestry University, Kunming 650000, China

**Keywords:** *Morinda citrifolia*, scopoletin, *COMT*, *CCoAOMT*, Iso-Seq

## Abstract

Scopoletin, the main component of clinical drugs and the functional component of health products, is highly abundant in noni fruit (*Morinda citrifolia*). Multiple enzyme genes regulate scopoletin accumulation. In the present study, differentially expressed genes of noni were analyzed by RNA sequencing (RNA-Seq) and the full-length genes by isoform-sequencing (Iso-Seq) to find the critical genes in the scopoletin accumulation mechanism pathway. A total of 32,682 full-length nonchimeric reads (FLNC) were obtained, out of which 16,620 non-redundant transcripts were validated. Based on KEGG (Kyoto Encyclopedia of Genes and Genomes) annotation and differential expression analysis, two differentially expressed genes, caffeic acid 3-O-methyltransferase (*COMT*) and caffeoyl-CoA O-methyltransferase (*CCoAOMT*), were found in the scopoletin accumulation pathway of noni. Real-time quantitative polymerase chain reaction (q-PCR), phylogenetic tree analysis, gene expression analysis, and the change in scopoletin content confirmed that these two proteins are important in this pathway. Based on these results, the current study supposed that *COMT* and *CCoAOMT* play a significant role in the accumulation of scopoletin in noni fruit, and *COMT* (gene number: gene 7446, gene 8422, and gene 6794) and *CCoAOMT* (gene number: gene 12,084) were more significant. These results provide the importance of *COMT* and *CCoAOMT* and a basis for further understanding the accumulation mechanism of scopoletin in noni.

## 1. Introduction

Noni (*Morinda citrifolia* Linn.) is an evergreen shrub growing in tropical and subtropical areas [1,2]. At the early stage of fruit formation, the surface remains rough. However, when the fruit ripens, the surface becomes smooth. The fruit is dark green when young, pale green to white when ripe, and soft and transparent grayish brown when fully mature [3].

For a long time, noni was considered a medicinal and edible plant by people in many tropical regions [4,5,6,7,8]. Previous studies confirmed that noni contains various functional phytochemicals used clinically as adjuvant medicine in hypertension, hyperglycemia, and cancer [9,10,11]. Scopoletin is one of the important functional phytochemicals of noni [12]. In addition, scopoletin is also found in a variety of plants such as *Arabidopsis thaliana* [13], *Chenopodium murale* [14], *Canscora decussata* [15], *Hypochaeris radicata* [16], and *Fagraea ceilanica* [17]. The metabolic pathway of scopoletin biosynthesis involves various types of chemical reactions and the catalysis of various enzymes. Glucose and lignin-derived aromatics are used as substrates, tyrosine and phenylalanine are produced by the shikimate acid pathway, phenylalanine ammonia-lyase (PAL) catalyzes phenylalanine to cinnamic acid, which is catalyzed into coumaric acid by cinnamate-4-hydroxylase (C4H), and tyrosine directly generates p-coumaric acid under tyrosine ammonia-lyase (TAL) catalysis. P-coumaric acid is converted to caffeic acid by O hydroxylated of 4-hydroxyphenyl acetic acid 3-hydroxylase A (HHA) or coumarate-3-hydroxylase (C3H), and caffeic acid is further methylated by caffeoyl-CoA O-methyltransferase (CCoAOMT) to produce ferulic acid. The formation of feruloyl-CoA is catalyzed by 4-coumarate CoA ligase (4CL) or feruloyl-CoA synthase (FCS). Feruloyl-CoA 6’-hydroxylase (F6′H) or coumaroyl CoA 2’-hydroxylase (C2’H) can catalyze the o-hydroxylation and subsequent spontaneous reactions (isomerization and endoesterification) of feruloyl-CoA, resulting in the formation of scopoletin [18,19]. Moreover, the role of CCoAOMT has been demonstrated in *Arabidopsis thaliana*. CCoAOMT promotes the soluble glucose-oleoconjugate in Arabidopsis leaves [20], which is crucial for scopoletin biosynthesis [21]. Furthermore, the accumulation of coumarins in *ccoaomt1* loss-of-function mutants is impaired [21,22]. Moreover, numerous studies have now also revealed that the main enzymes involved in scopoletin synthesis were 4CL, COMT, CCoAOMT, and F6H [23,24,25,26]. The preferred substrates of COMT are caffeoyl aldehyde and 5-hydroxyconiferaldehyde, but other Class II OMTs catalyzed the methylation of flavonoids, flavonols, phenylpropylene, and phenols [27].

We determined the genome size of noni by FCM (flow cytometry) previously, and the results show that the genome size of noni was 1.15 to 1.16 GB (the detailed measurement results are shown in Supplementary Figure S1). Noni’s large genome limits the important secondary metabolite biosynthesis pathway’s elucidation, while RNA-Seq can help elucidate the dynamics of fruit development. Yu et al. studied the dynamic changes in soluble sugars in plum fruits at different developmental stages by RNA-Seq. The result shows that among the transcripts identified as encoding key enzymes of soluble sugar metabolism and accumulation, some genes regulate the accumulation of fructose at the green stage, while others regulate the degradation of sucrose at the early stage of fruit development. Even one gene plays a different role at different stages of development [28]. By combining the transcriptome and metabolome sequencing results, a study found that glycerophospholipid metabolism was closely related to post-harvest softening of pear, and the differential genes that were significantly enriched in the KEGG pathway such as glycerophospholipid metabolism and fatty acid degradation were identified, which laid a foundation for subsequent research [29].

In our previous study, we found that scopoletin content showed regular dynamic changes during post-ripening in noni fruit, which aroused our interest [30]. For RNA-Seq, fresh analysis materials are required so that RNA can be extracted for analysis. However, in the preliminary experiment, it was found that 48 h after post-ripening, the RNA degradation of noni fruit was serious and transcriptome analysis was not possible. So, in this study, dynamic changes of 0–48 h were selected to reflect the transcriptional regulation and substance metabolism during the post-ripening of noni fruit.

Iso-Seq is based on the PacBio Sequel third-generation sequencing platform, which directly obtains complete transcripts containing 5 ‘UTR (untranslated region), 3′ UTR, and poly(A) tails without breaking the splicing, to accurately analyze structural information such as variable splicing and fusion genes of reference genome species. Moreover, Iso-Seq overcomes the problem of short splicing and incomplete information of non-reference genome species [31,32]. It directly generates full-length transcripts and further optimizes the genome annotation file, which provides an opportunity to discover new genes and subtypes and to study the complexity of the plant transcriptome [33]. Studies have shown that the combination of RNA-Seq and Iso-Seq can accurately quantify the abundance of transcripts [34].

Therefore, in this study, the root, stem, leaf, and fruit of noni were used for Iso-Seq, and full-length transcripts were obtained. To explore the physiological and molecular changes of noni fruit after harvest, RNA-Seq was performed on noni fruit. Two key genes, COMT and CCoAOMT, were screened in the accumulation process of scopoletin in noni fruits after harvest by combined analysis of the RNA-Seq and Iso-Seq. Then, the changes in gene expression and scopoletin content within 48 h were measured to preliminarily verify the importance of these two genes.

## 2. Methods and Materials

### 2.1. Plant Material Preparation

The noni used in this study was introduced from Hawaii, the USA, in 2007 and grown in the planting base in Yuanjiang County, Yunnan Province, China, where the latitude is between 23°19′ and 23°55′, the longitude is between 101°39′ and 102°22′, the maximum altitude is 2580 m, and the minimum altitude is 327 m. Dr. Tian Wu undertook the formal identification of the plant material used in our study. Yuanjiang is a dry-hot valley with an average temperature of the hottest month of 16 °C–29 °C, the average temperature of the coldest month of 7 °C–17 °C, and the average annual temperature of 29 °C. the annual average humidity in Yuanjiang is 71%; the annual average precipitation is 770–2400 mm. The pH of the red soil there is 6.0–6.5. Thus, the climatic conditions are suitable for the growth of noni. This study was conducted at the Southwest Landscape Architecture Engineering Research Center of State, China, from 3 to 27 November 2019. Freshly harvested fruits and the fruits stored for two days at 25 °C and 70% relative humidity were sampled, with three biological replicates per sample. In this study, the material used for RNA-Seq was noni fruit. Three trees in the base were randomly selected, and the harvested noni fruits were divided into two groups (0d and 2 d after harvest), with at least three noni fruits in each group. Using these materials, six Illumina RNA-Seq libraries were constructed. As with RNA-Seq, 3 trees were randomly selected from the base for sampling, and the root, stem, leaf, and fruit of noni were collected for Iso-Seq. Mixing different tissues from each tree into one sample yielded three biological replicates. The length of harvested fruits for the current study was about 5 cm. The samples were rapidly frozen with liquid nitrogen and stored in a −80 °C refrigerator. The experimental research and field studies on plants comply with relevant institutional, national, and international guidelines and legislation. The plants were collected, and we ensured permission to collect the noni plant.

### 2.2. RNA Extraction and Quality Assessment

Total RNA was extracted from the tissue using TRIzol^®^ Reagent according to the manufacturer’s instructions (Invitrogen, Carlsbad, CA, USA), and genomic DNA was removed using DNase I (TaKara, Beijing, China). Then, Nanodrop was used to detect RNA purity (OD_260/280_ ratio), Qubit accurately quantified RNA concentration, and Agilent 2100 accurately detected RNA integrity. Finally, a high-quality RNA sample (OD_260/280_ = 1.8~2.2, OD_260/230_ ≥ 2.0, RIN ≥ 6.5, 28 S:18 S ≥ 1.0, >10 μg) was used to construct a sequencing library.

### 2.3. PacBio Iso-Seq Library Preparation and Sequencing

Oligo (dT) was used to enrich the mRNA that contains polyA, and then mRNA was reverse-transcribed to cDNA by using SMARTer PCR cDNA Synthesis Kit (Takara, Beijing, China). PCR amplification enriched the synthesized cDNA, and the optimal PCR conditions were determined by cyclic optimization. Part of the cDNA was screened for fragments with BluePippin to enrich for fragments above 4 Kb, and the screened fragments were subjected to large-scale PCR to obtain sufficient total cDNA. Full-length cDNA was used for damage repair, end repair, the connection of the SMRT (Single Molecule Real-Time) dumbbell connector, and construction of the molar mixed library with unscreened fragments and fragments larger than 4 Kb. Exonuclease digestion removed sequences of unconnected joints at both ends of cDNA, and finally, primers were combined and DNA polymerase was bound to form a complete SMRT bell library.

### 2.4. Illumina RNA-Seq Library Preparation and Sequencing

The library was constructed with 5 μg total RNA. After the isolation of mRNA by magnetic bead separation, the mRNA was broken by ions. The double-stranded cDNA was synthesized, toned 3′ plus A, and connected to the index connector (TruseqTM RNA sample prep Kit, Illumina, San Diego, CA, USA). Afterward, the target fragments were purified for library enrichment by 15 cycles of PCR amplification and agarose electrophoresis at a concentration of 2%. Samples were then quantified using TBS380 (Picogreen, Invitrogen, Carlsbad, CA, USA) and mixed in proportion to the data and operated on the computer. Next, bridge PCR amplification was performed on the cBot to generate clusters. Finally, 2*150 bp were sequenced using the platform of Illumina Hiseq.

### 2.5. Iso-Seq Data Analysis

After PacBio sequencing was completed, the original disconnecting and low-quality reads were carried out. The output was filtered and processed with SMRTlink v5.1 software, and the final data obtained were considered valid. Self-correcting subreads formed CCS (Circular Consensus Sequence) to obtain high-quality transcriptional consistent sequences. The nonchimeric consistent sequences containing 5′ primer, 3′ primer, and poly A tail are called full-length nonchimeric sequences (FLNC). Since there are a large number of redundant sequences in the FLNC, the redundant sequences need to be clustered together to remove the redundancy by the ICE algorithm [35] and obtain corrected consensus sequences.

### 2.6. Illumina RNA-Seq Data Quality Control

In this study, the software used for quality control was trimmomatic (http://www.usadellab.org/cms/uploads/supplementary/Trimmomatic, accessed on 20 September 2018). First, the joint sequences in reads and the reads without inserted fragments due to the self-connection of the joint were removed. Then, bases with lower quality (mass value less than 20) at the end of the sequence (3 ‘end) were trimmed. If there were bases with a mass value of less than 10 in the rest of the sequence, the whole sequence was removed. In addition, reads with an N ratio of more than 10% were removed. Finally, the sequence with a length of less than 75 bp after quality trimming was discarded, and high-quality sequencing data (clean data) were obtained.

### 2.7. Transcript Correction and Redundancy Removal

The disadvantage of Iso-Seq is that the additional insertion of bases leads to a higher frequency of errors, especially in the homopolymer region. However, such errors occurred randomly and did not have the sequencing bias as the RNA-Seq. The drawback of RNA-Seq is that the read length is short and splicing errors may occur. So, the high-accuracy sequences of RNA-Seq can be used to calibrate the sequencing result of Iso-Seq further. With the RNA-Seq data available, Lordec [36] was used to correct further the polished consensus sequences with the RNA-Seq data. The CD-HIT [37,38] was used to cluster and compare nucleic acid sequences through sequence alignment, and the redundant and similar sequences were removed. We clustered the corrected transcript sequences according to the 95% similarity between sequences and conducted statistics on the distribution of length and frequency before and after the redundancy of the transcript.

### 2.8. Gene Functional Annotation

The following databases were used to annotate the gene functions: NCBI Non-redundant Protein (Nr) (https://www.ncbi.nlm.nih.gov/protein/, accessed on 25 September 2018), Gene Ontology (GO, http://www.geneontology.org/, accessed on 25 September 2018), euKaryotic Ortholog Groups (KOG, ftp://ftp.ncbi.nih.gov/pub/COG/KOG/, accessed on 25 September 2018), Swiss (https://www.uni-prot.org/uniprot/, accessed on 25 September 2018), Pfam (https://pfam.xfam.org, accessed on 25 September 2018), and Kyoto Encyclopedia of Genes and Genomes (KEGG, http://www.kegg.jp/, accessed on 25 September 2018).

### 2.9. Differential Expression Analysis

In RNA-Seq analysis, gene expression levels were calculated by the number of clean reads in the genomic region. Then, reads obtained by sequencing were mapped with Iso-Seq full-length transcripts. According to the mapping result between all RNA-Seq and Iso-Seq transcripts, the RPKM (Reads Per Kilobase per Million mapped reads) value of each transcript in the samples was calculated, and the resulting value was used as the expression amount of the transcript. Finally, the expression of the transcripts in the samples of each group was analyzed for significant differences, and the relative differentially expressed transcripts were identified and visualized. The significance of expression difference between samples is generally considered to be less than 0.05, and the q value was the corrected *p* value with greater statistical significance. The RPKM value was the standard to measure gene expression level, and the expression difference analysis software was edgeR (10.18129/B9.bioc.edgeR, accessed on 12 October 2018). RSEM (http://deweylab.biostat.wisc.edu/rsem/, accessed on 12 October 2018) [39] was used to quantify gene and isoform abundances. So, in this study, a q value of less than 0.05 was used to perform Go enrichment analysis to identify which DEGs (different expression genes) were significantly enriched in GO terms and metabolic pathways at Bonferroni-corrected *p*-value ≤0.05 compared with the whole-transcriptome background. GO functional enrichment and KEGG pathway analysis were carried out using Goatools (https://github.com/tanghaibao/Goatools, accessed on 12 October 2018) and KOBAS (http://kobas.cbi.pku.edu.cn/home.do, accessed on 12 October 2018) (Mark et al. 2010).

$$\mathrm{RPKM}=\frac{tatal\_exon\_reads}{mapped\_reads(millions)*exon\_length(KB)}$$

### 2.10. Phylogenetic Analysis of CCoAOMTs and COMTs

Based on the functional annotation of KEGG and the transcriptome sequencing analysis of fresh and two-day ripened fruit, some genes were differentially expressed in the scopoletin pathway. Two differentially expressed genes *COMT* and *CCoAOMT* were identified in the pathway related to the synthesis of ferulic aldehyde, a precursor synthesized by scopoletin. Then, eight *COMTs* and four *CCoAOMTs* in noni were found in the annotation library of Iso-Seq. Two phylogenetic trees were constructed based on 42 *CCoAOMT* nucleotide sequences and 30 *COMT* nucleotide sequences that covered different species to analyze evolutionary relationships and predict the gene function. Accession numbers of sequences used in this article are listed in Supplementary Tables S1 and S2. These sequence data can be found in the Phytozome and GenBank/EMBL data libraries. The nucleotide sequences of the noni *CCoAOMTs* and *COMTs* used in the corresponding phylogenetic trees are shown in Supplementary Table S3.

### 2.11. q-PCR

Noni fruit RNA was extracted with an RNA kit (Vazyme Biotech co., Itd., Nanjing, China), with 450 ng RNA as a template, and a reverse transcriptase kit (Thermo Scientific™ EP0733, Santa Clara, CA, USA) was used for reverse transcription. The reverse transcriptase reaction was carried out at 25 °C for 10 min, then at 50 °C for 30 min, and finally at 85 °C for 5 min after heating. The obtained cDNA was used as a template for q-PCR amplification using gene-specific primers and SG Fast q-PCR Master Mix (High Rox, Sangon Biotech (Shanghai) Co., Ltd., Beijing, China). Each sample was analyzed three times and standardized with Actin as an internal control. The primers used in the experiment are shown in Supplementary Tables S4 and S5. According to the manufacturer BioRad (Santa Clara, CA, USA), to provide q-PCR application, the dissolution curve (from 65 °C to 95 °C) was analyzed, and 2 ^−ΔΔ^ Ct was used for evaluation-related gene expression. The q-PCR conditions were: reaction at 95 °C for 3 min, reaction at 95 °C for 7 s, reaction at 57 °C for 10 s, and reaction at 72 °C for 15 s. Three independent biological replicas estimated the mean and standard errors. q-PCR amplification efficiency was measured for each gene to ensure the reliability of the result.

### 2.12. Treatment of Noni Fruit and Scopoletin Content Determination

Ripe noni fruit (cream-colored) with a constant size was selected randomly. Then, the noni fruit was stored at the temperature of 25 °C and sampled at 0 h, 2 h, 12 h, 24 h, and 48 h. The methods of sample processing and determination of scopoletin content were consistent with the previous description [30]. 

## 3. Results

### 3.1. Transcriptome Sequencing and Annotation

RNA from freshly harvested fruits and the fruits stored for two days were extracted for RNA-Seq and produced six Illumina RNA-Seq clean reads (Supplementary Table S6). Then, Trinity (http://trinityrnaseq.sourceforge.net/trinityrnaseq-r2013-02-25, accessed on 20 September 2018) was used to splice the short sequence produced by RNA-Seq, and then 35,721 unigenes were obtained.

RNA from root, stem, leaf, and noni fruit was mixed for Iso-Seq. A total of 25,575,577 raw subreads (35.04 G) with an average read length of 1370 bp were obtained. After removing adapters and artifacts, 483,741 CCSs were obtained in total. Then, 362,241 full-length (FL) reads and 32,682 full-length nonchimeric reads (FLNC) were obtained with SMRTlink to detect the 5′-primer, 3′-primer, and poly-A tails.

Lordec was used to correct the high error rate of Iso-Seq in the polished consensus sequence based on the Illumina RNA-Seq reads. Then, CD-HIT was used for sequence alignment and clustering, and the corrected transcript sequences were clustered to remove redundancy according to the 95% similarity between the sequences. In total, the current study obtained 32,600 polished consensus reads and 16,620 transcripts.

To ensure structural accuracy, sequence integrity, and sequence expression accuracy, the results of RNA-Seq and Iso-Seq were combined for further analysis. In the analysis, the sequenced reads were compared with three generations of full-length transcripts, and the mapping ratio was obtained (Supplementary Table S7).

**Figure 1:**
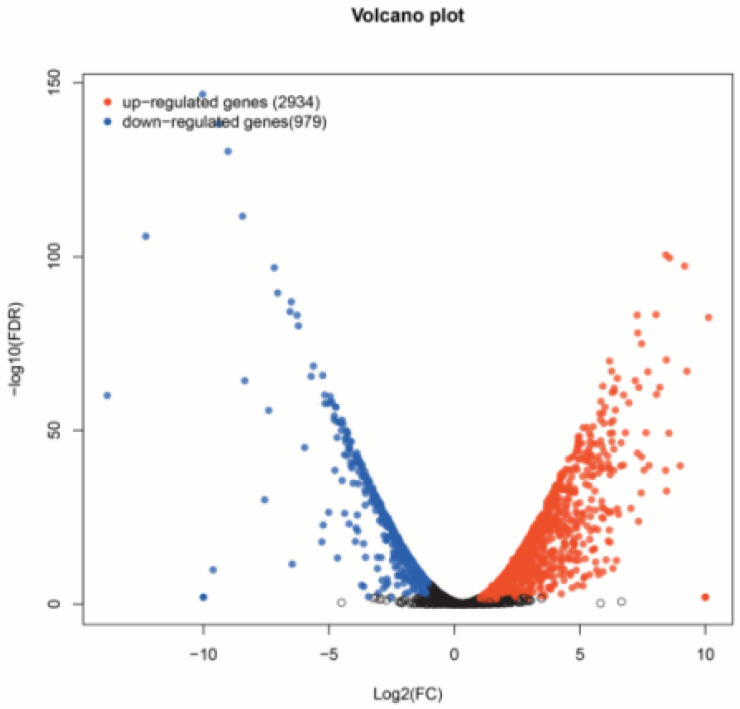
Differential gene visualization scatter plot. Red dots indicate significantly up-regulated genes, blue dots indicate significantly down-regulated genes, and black dots indicate non-significantly different genes. The closer the point is to 0, the lower the expression level is; the more the point is off the diagonal, the greater the expression difference of the gene or transcript between the two samples is.

**Figure 2:**
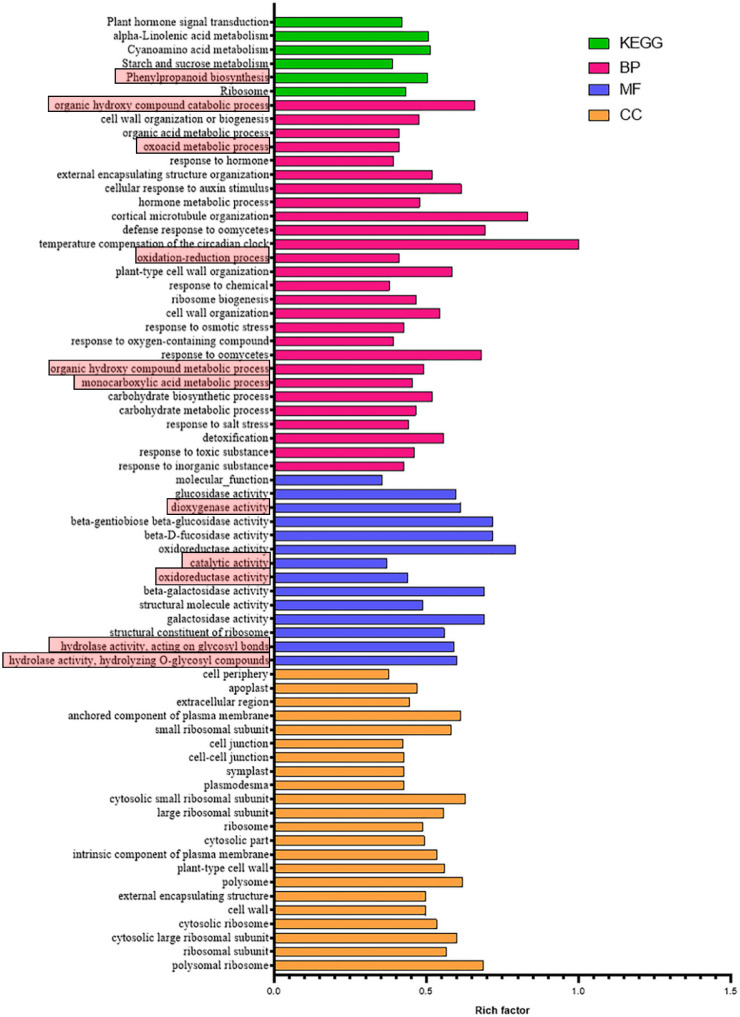
The corrected *p* value of less than 0.05 of KEGG enrichment analysis and GO enrichment of DEGs in the group of noni fresh fruit and noni fruit harvest after 2 days. Rich factor = gene ratio/background ratio (gene ratio: DEGs in the term, background ratio: all genes in the term).

**Figure 3:**
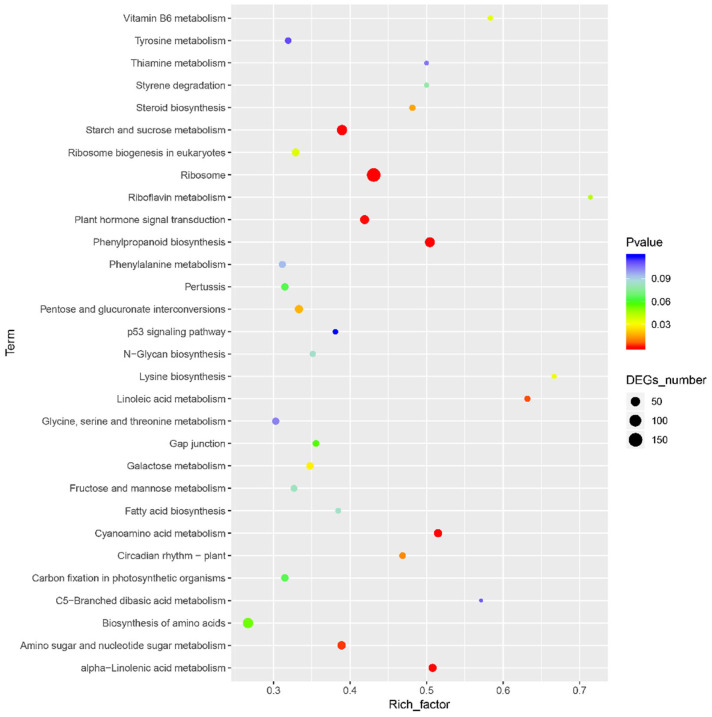
Enrichment of KEGG. The horizontal axis represents the enrichment factor, that is, the ratio of the number of differential genes enriched to a certain KEGG term to the number of background genes obtained by sequencing. The ordinate represents the function enriched in this KEGG term: the larger the circle, the more differential genes enriched in this function. The color spectrum from blue to red represents the uncorrected *p*-value.

Based on the mapping results between PacBio and Illumina data, the RPKM value was the standard to measure gene expression level, and a *p*-value (probability of hypothesis testing by statistical models) of less than 0.05 was identified as DEGs. A total of 3913 differentially expressed genes were found among all 11,281 single genes; 2934 were up-regulated, and 979 were down-regulated (Figure 1, Supplementary Tables S8 and S9). Then, the *p*-value was corrected by multiple hypothesis testing, and genes with a corrected *p* value of less than 0.05 were obtained to perform GO enrichment analysis and KEGG pathway analysis (Figure 2). These genes were categorized into biological processes (BPs), cellular components (CCs), and molecular functions (MFs). In MF classification, DEGs are involved in hydrolase activity, oxidoreductase activity, catalytic activity, and dioxygenase activity. In BP classification, DEGs are involved in the organic hydroxy compound catabolic process, oxoacid metabolic process, oxidation–reduction process, organic hydroxy compound metabolic process, and monocarboxylic acid metabolic process. These reactions are related to secondary metabolism in plants and are highlighted by red boxes in the figure. In addition, KEGG analysis showed that most of the differential genes were enriched in the metabolic pathway of phenylpropanoid biosynthesis, and this pathway contains scopoletin synthesis. Among these DEGs, some of them are involved in phenylpropanoid biosynthesis and phenylalanine metabolism, which is also the first step of the biosynthesis of scopoletin (Figure 3).

### 3.2. Transcriptome Data Were Verified by q-PCR

To verify the accuracy of transcriptome sequencing, 10 up-regulated and 10 down-regulated genes were selected for q-PCR. The q-PCR result is consistent with that of transcriptome sequencing, indicating the accuracy of transcriptome data (Supplementary Figure S2).

### 3.3. COMT and CCoAOMT Play a Key Role in the Accumulation of Scopoletin

**Figure 4:**
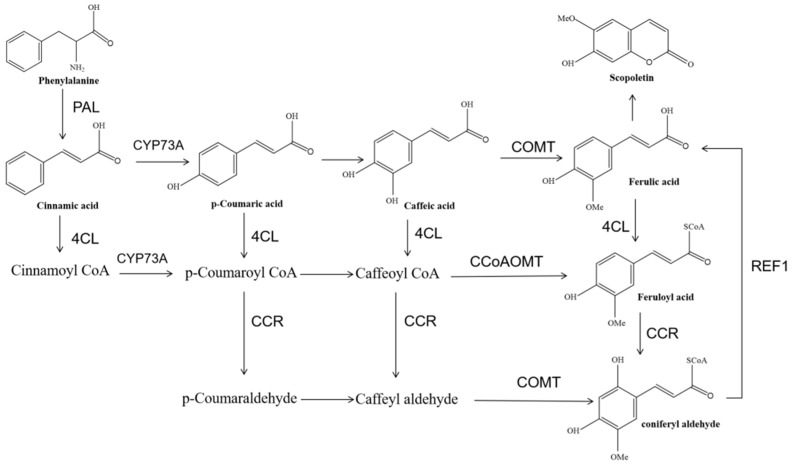
Putative scopoletin biosynthetic pathway. PAL: phenylalanine ammonia-lyase. 4CL: 4-coumarate--CoA ligase. CYP73A: trans-cinnamate 4-monooxygenase. CCR: cinnamoyl-CoA reductase. COMT: caffeic acid 3-O-methyltransferase. CCoAOMT: caffeoyl-CoA O-methyltransferase. REF1: coniferyl-aldehyde dehydrogenase.

Based on the strong scopoletin functional activity in previous studies [30], the involvement of the scopoletin accumulation pathway in the phenylpropyl pathway attracted our attention. In the scopoletin accumulation pathway, scopoletin is produced from phenylalanine by a series of enzymatic reactions. In this pathway, the related genes are *PAL*, *CYP73A*, *4CL*, *COMT*, *CCoAOMT*, *REF1*, and *CCR* (Figure 4). Only all members of *COMT* and *CCoAOMT* showed up-regulated and different expressions (the result is shown in Supplementary Table S10), and q-PCR was used to verify the importance of these two genes in noni.

**Figure 5:**
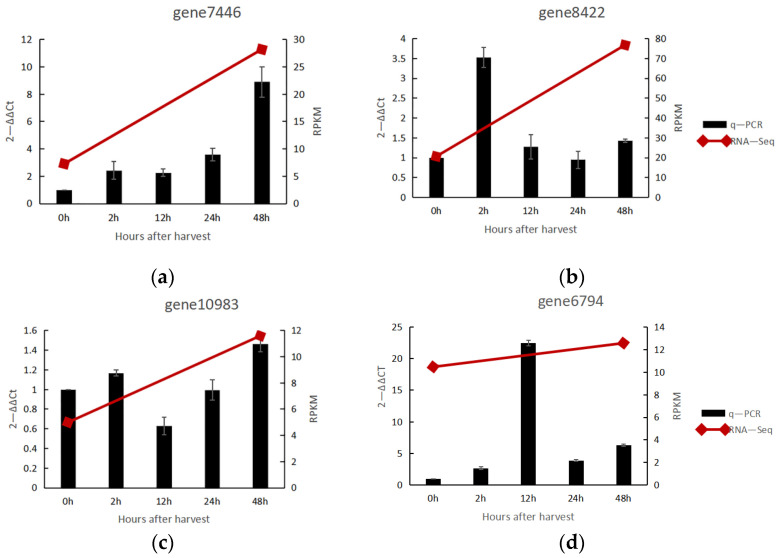
Relative gene expression of *COMT* in noni fruit. (**a**) Relative expression of gene 7446; (**b**) Realtive expression of gene 8422, (**c**) Realtive expression of gene 10,938, (**d**) Realtive expression of gene 10,938.

Both groups of genes fall under the OMT group and most commonly share the AdoMet MTases domain. However, *COMT* has a unique dimerization domain, which *CCoAOMT* lacks. In addition, the molecular weight of *COMTs* is in the range of 39–40 kDa, whereas that of *CCoAOMTs* is in the range of 27–29 kDa [40]. COMT O-methylates at the C5 position of the phenolic ring, and the O-methylation at the C3 position of the phenolic ring involves the CCoAOMT [20,41]. Eight unigenes (gene number: gene 4888, gene 6794, gene 7263, gene 7446, gene 10,983, gene 6862, gene 8422, gene 12,081) were annotated as *COMT* in the full-length sequence obtained by Iso-Seq. Moreover, combined RNA-Seq and Iso-Seq data showed two genes (gene number: gene 7446 and gene 8422) with high differential expression and two genes (gene number: gene 10,983 and gene 6794) with low differential expression. So, these genes were selected for q-PCR verification, and the results are shown in Figure 5. These genes were expressed in a consistent trend of RNA-Seq, and all reached a significant difference within 48 h after harvest. Except for the weak expression of gene 10,983, the expression of the other three genes was significant. The relative expression of gene 8422 reached 3.5-fold at 2 h after harvest. For this phenomenon, the current study proposed that this gene was the first responsive among these four genes. The relative expression of gene 6794 reached 22.5-fold at 12 h after harvest, and gene 7446 reached 8.9-fold at 48 h after harvest. The timing of the soar in the expression of these three genes was sequential, which made us consider it a plant energy-saving mechanism.

**Figure 6:**
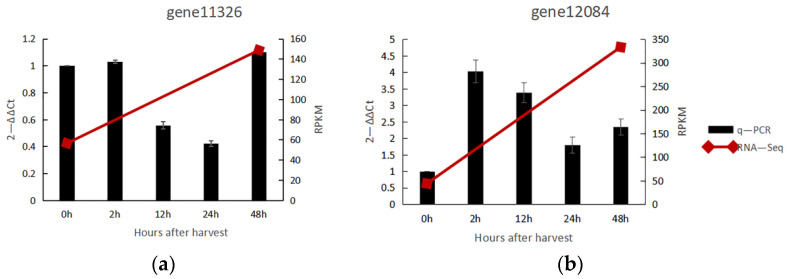
Relative gene expression of *CCoAOMT* in noni fruit. (**a**) Relative expression of gene 11,326; (**b**) Realtive expression of gene 12,084.

Two unigenes (gene number: gene 11,326 and gene 12,084) were annotated as *CCoAOMT* in the full-length sequence obtained by Iso-Seq. The expression of these two genes in the two days after the natural ripening of noni fruit was verified by q-PCR, and the results are shown in Figure 6. Their expression results are consistent with the RNA-Seq, showing average up-regulated expression, with gene 12,084 showing relatively high up-regulated expression, 4-fold at the 2 h after harvest.

### 3.4. Phylogenetic Analysis of McCCoAOMTs and McCOMTs

**Figure 7:**
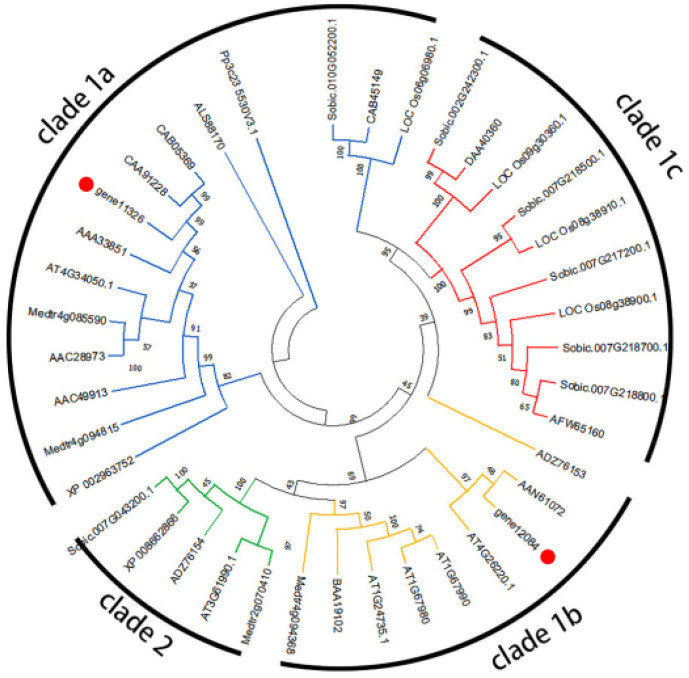
Phylogenetic tree of *McCCoAOMT* (gene 12,084 and gene 11,326) and other previously characterized plant *CCoAOMTs*. Numbers at branch points are bootstrap values representing the confidence level as a percentage based on 1000 repeats.

**Figure 8:**
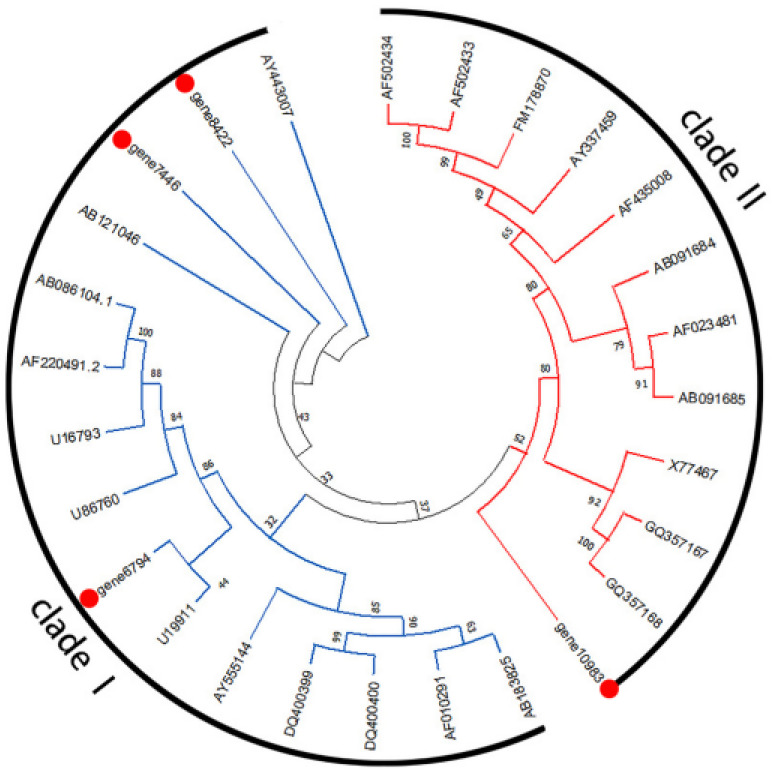
Phylogenetic tree of *McCOMT* (gene 10,983, gene 7446, gene 8422, and gene 6794) and other previously characterized plant *COMTs*. Numbers at branch points are bootstrap values representing the confidence level as a percentage based on 1000 repeats.

The result of phylogenetic relationships (Figure 7) indicates that the *CCoAOMTs* could be classified into four clades (1a, 1b, 1c, and 2). Gene 11,326 was grouped in clade 1a, and gene 12,084 was grouped in clade 1b. The result of phylogenetic relationships (Figure 8) indicates that the *COMTs* could be classified into two clades (clade I and clade II). Gene 10,983 was grouped in clade II, and gene 7446, gene 8422, and gene 6794 were grouped in clade I.

### 3.5. Accumulation of Scopoletin in Noni Fruit

**Figure 9:**
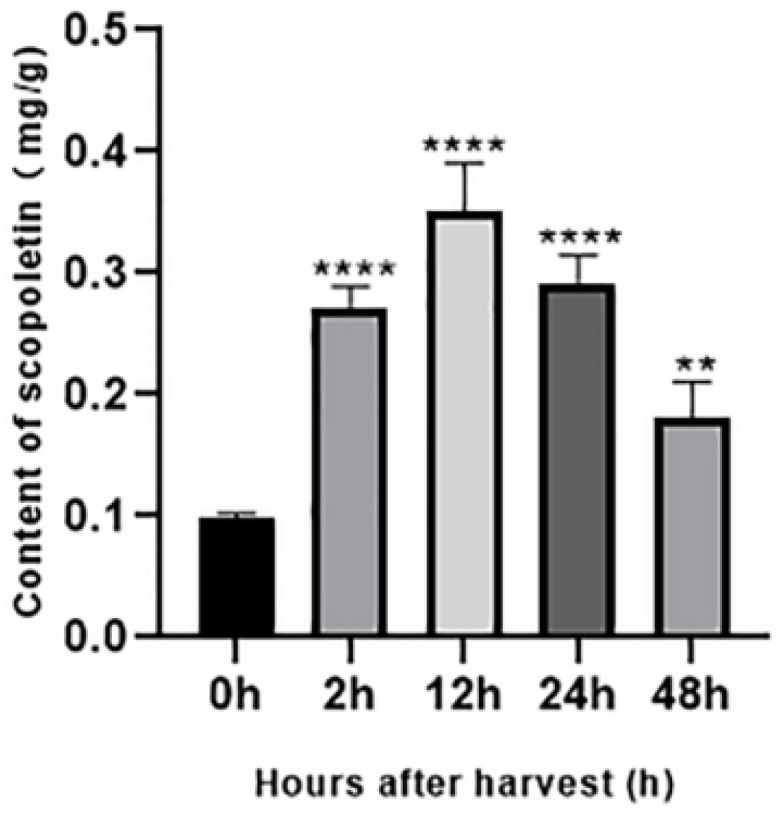
Change in the content of scopoletin in noni fruit within 48 h after harvest. Data are presented as the mean ± S.D. of three individual experiments, performed in triplicate. *p* values of less than 0.05 were considered significant. Note: ** indicates *p* less than 0.01 and **** indicates *p* less than 0.0001.

To further prove the important role of *COMT* and *CCoAOMT* in the accumulation of scopoletin in noni fruit from the material level, we determined the scopoletin content in noni fruit within 48 h after harvest. As shown in Figure 9, the content of scopoletin in noni fruit increased rapidly and significantly in the second hour after harvest and then reached the maximum value of 0.35 mg/g at the 12th hour. After 12 h of the noni fruit harvest, the scopoletin content began to show a downward trend, and the content of scopoletin in noni fruit was 0.18 mg/g at the 48th hour after harvest. The scopoletin content of noni fruit at the 48th hour was half of the 12th hour, but that was double the 0 h, and the difference was significant.

The content of scopoletin in noni fruit significantly increased in a short time and then decreased. However, the general trend increased gradually with the extension of post-ripening. These results suggest that in the short time after harvest, the change in scopoletin content rapidly responded to the regulation of key genes in its synthesis pathway to some extent. The results of q-PCR suggest that different copies of the same gene are expressed in some order, to achieve the purpose of energy saving. For example, scopoletin increased rapidly in the second hour, which might be due to the simultaneous up-regulation of four copies of *COMT*. At the 12th hour, although scopoletin continued to increase, only gene 6794 was strongly up-regulated, and the expression of the other three genes decreased; gene 10,983 was even down-regulated, which might be performing some kind of gene saving program.

## 4. Discussion

Natural biochemicals are an important source of drug discovery [42]. Noni, which has the secondary metabolite scopoletin, has attracted research attention as one of the adjuvant drugs in clinical treatment. Therefore, elucidating the biosynthesis pathway will lay a foundation for increasing the supply of this important substance.

Fruit still has to undergo a series of physiological changes, such as ripening, aging, and death after harvest, and still changes the internal material through respiration. Previous studies showed that in the process of fruit ripening, the metabolism of reactive oxygen species was strengthened, which would produce a toxic effect on cells and lead to the destruction of cell membrane structure [43]. Therefore, during the process from fresh fruit to two days after harvest, the fruit of noni was still undergoing complex physiological activities. In previous studies, the scopoletin content increased in the fruit two days after harvest compared with the fresh fruit of noni [30]. It was found that a large number of phenylpropanoid metabolic pathway genes were highly expressed in plants under biotic and abiotic stress, resulting in the accumulation of a variety of secondary metabolites, including scopoletin [44]. Scopoletin synthesis involves the first catalytic steps of the phenylpropanoid pathway, leading to p-coumaric acid [45]. Therefore, active phenylpropanoid metabolism will provide a sufficient precursor for the accumulation of scopoletin.

The current study hypothesized a differential expression of the enzyme gene related to the accumulation of scopoletin. Through transcriptome analysis, phylogenetic tree analysis, and q-PCR verification, the current study determined that *McCOMT* and *McCCoAOMT* played an important role in the accumulation of scopoletin in noni. *McCOMT* and *McCCoAOMT* are in the phenylpropanoid pathway, and all enzymes in the pathway were characterized, and in many cases, gene regulation is at the transcriptional level.

The preferred substrates of COMT are caffeoyl aldehyde and 5-hydroxyconiferaldehyde [25,46]. According to the annotation of metabolic pathways in transcriptome sequencing, it is known that in the accumulation of scopoletin in noni, the transformation from caffeic acid to ferulic acid was realized. Thus, COMT could catalyze the conversion of coffee acid to ferulic acid. The transgenic tobacco plants with down-regulated *COMT* gene expression demonstrate that COMT plays a crucial role related to controlling lignin and phenol content in plants. Moreover, COMT activity may be related to flavonoid production in the plant lignin pathway [47]. For this, studies have shown that plants strengthen their cell walls through the accumulation of phenolic compounds, which was thought to be a ubiquitous defense response [48]. After the RNA-Seq, Iso-Seq, phylogenetic tree analysis, and the q-PCR validation of differentially expressed genes in our study, we found that the three copies of *McCOMT* (gene number: gene6794, gene8422, and gene7446) were regulated obviously, which was similar to the *COMTs* of tobacco that has certain genes that would be regulated. The phylogenetic tree analysis of *McCOMT* was grouped into two clades, and gene 7446, gene 8422, and gene 6794 were grouped in clade I which are active for phenolic compounds involved in lignin biosyntheses such as caffeic acid and 5-hydroxyferulic acid and their respective aldehydes and alcohols. Moreover, it was found that class I included flavonoid, simple phenol, and multifunctional *COMT* genes in Populus, and these *COMTs* are regulated to defend against biotic and abiotic stresses [49]. It was found that the ripening process of blueberry fruit was also accompanied by the phenomena of fruit softening. *COMT* was also found to be differentially expressed, and *VcCOMT38*, *VcCOMT57*, *VcCOMT40*, and *VcCOMT92* belonged to clade I and are regulated consistently with those of lignin during fruit development [50]. However, gene 6794, gene 8422, and gene 7446 show no consistent up-regulation, which may indicate gene redundancy. Similarly, functional redundancy has also been reported in this gene family [51].

CCoAOMT could catalyze the methylation of acyl-coenzyme, and the function has been detected in a variety of other plants [52]. CCoAOMT has been shown to successfully methylate various flavonoids, caffeoyl CoA, anthocyanins, coumarins, and aromatic esters [53,54]. Consistent with the results of previous research, the phylogenetic tree analysis of noni *McCCoAOMT* was grouped into four clades, and gene 12,084 was grouped in clade 1b that was involved in the biosynthesis of some metabolites, such as flavonoids and phenylpropanoids [55]. In addition, the phenomenon of fruit softening was also observed after the harvest of noni fruits, and fruit softening was a significant manifestation of reduced lignin content [56]. Many research studies have confirmed that CCoAOMT is a methyltransferase that plays an important role in lignin biosynthesis. The down-regulation of the *CCoAOMT* in different plant species resulted in significant decreases in lignin contents. In contrast, the overexpression of this gene led to an increase in the lignin content, indicating the essential role of this gene in the process of lignin biosynthesis [57].

Increasing studies have confirmed the relationship between *COMT* and *CCoAOMT* and the synthesis of scopoletin in recent years. *CCoAOMT1* in *Arabidopsis thaliana* contributed to the formation of soluble sinapoyl conjugates in leaves and was crucial for the accumulation of coumarin scopoletin [58]. Feruloyl CoA was a key precursor in scopoletin accumulation, and COMT and CCoAOMT were the key enzymes in the synthesis of ferulic acid [59]. In this study, two copies of *McCCoAOMT* were found up-regulated, and the current study preliminarily believed that *McCCoAOMT* was another key gene for the synthesis of scopoletin in noni in combination with previous studies on the changes in scopoletin content in the post-maturation process of noni fruit.

## 5. Conclusions

After the above verification and analysis, we supposed that *McCOMT* and *McCCoAOMT* both played a significant role in accumulating scopoletin in the noni fruit. Among the four unigenes of *COMT*, we speculated that gene 7446, gene 8422, and gene 6794 were more important, while gene 12,084 of *CCoAOMT* was more important. Nevertheless, it needs to be further verified later. *McCOMT* needs to construct the protein vector of the corresponding target gene, and further study is needed on whether the function of the *McCOMT* is caffeic acid, caffeoyl aldehyde, coniferyl aldehyde, or just one of them based on protein purification later. In addition, based on the established noni genetic transformation system [60,61], the homologous transformation of *McCOMT* and *McCCoAOMT* can be performed to obtain the evidence of gene function directly. At that time, the accumulation pathway of scopoletin will be clearer.

## Data Availability

All data generated or analyzed during this study are included in this published article and its supplementary information files. The transcript assemblies have been deposited and are publicly available at NCBI with accession PRJNA503490. The Iso-Seq data have been deposited and are publicly available at NCBI with accession SRR12716286.

## Supplementary Materials

The following supporting information can be downloaded at: https://www.mdpi.com/article/10.3390/genes13111993/s1, Figure S1: Results of genome size determination; Figure S2: q-PCR verification of RNA-Seq (10 up regulated and 10 down regulated genes). Table S1: Gene ID used in the phylogenetic tree CCoAOMT, Table S2: Gene ID used in the phylogenetic tree COMT, Table S3: Nucleotide sequence of COMT and CCoAOMT, Table S4: q-PCR primers (up-regulated and down-regulated), Table S5: q-PCR primers of COMT and CCoAOMT, Table S6: Statistical table of RNA-Seq data after quality control, Table S7: Mapping ratio, Table S8: Annotation of Iso-Seq, Table S9: Different expression genes, Table S10: Related genes in scopoletin pathway.

## References

[1] Jahurul M., Patricia M., Shihabul A., Norazlina M., George M.R., Noorakmar A., Lee J., Jumardi R., Jinap S., Zaidul I. (2021). A review on functional and nutritional properties of noni fruit seed (*Morinda citrifolia* L.) and its oil. Food Biosci..

[2] Marisa M.W., Samuel M., Matthew S.S. (2018). Volatile changes in Hawaiian noni fruit, *Morinda citrifolia* L., during ripening and fermentation. J. Sci. Food Agric..

[3] Li J., Niu D.B., Zhang Y., Zeng X.A. (2020). Physicochemical properties, antioxidant and anti proliferative activities of polysaccharides from *Morinda citrifolia* L. (Noni) based on different extraction methods. Int. J. Biol. Macromol..

[4] Deng Y., Chin Y.W., Chai H., Keller W.J., Kinghorn A.D. (2007). Anthraquinones with quinone reductase-inducing activity and benzophenones from *Morinda citrifolia* (noni) roots. J. Nat. Prod..

[5] Assi R.A., Darwis Y., Abdulbaqi I.M., Khan A.A., Lim V., Laghari M. (2015). *Morinda citrifolia* (noni): A comprehensive review on its industrial uses, pharmacological activities, and clinical trials. Arab J Chem..

[6] Wang J.F., Qin X.C., Chen Z.Y., Ju Z.R., He W.J., Tan Y.H., Zhou X.J., Tu Z.C., Lu F.G., Liu Y.H. (2016). Two new anthraquinones with antiviral activities from the barks of *Morinda citrifolia* (Noni). Phytochem. Lett..

[7] West B.J., Deng S.X., Isami F., Uwaya A., Jensen C.J. (2018). The Potential Health Benefits of Noni Juice: A Review of Human Intervention Studies. Foods.

[8] Lohania M., Majrashib M., Govindarajulu M., Patel M., Ramesh S., Bhattacharya D., Joshi S., Fadan M., Nadar R., Darien B. (2019). Immunomodulatory actions of a Polynesian herb noni (*Morinda citrifolia* L.) and its clinical applications. Complement. Ther. Med..

[9] Thomaz D.V., Couto R.O., Oliveira Roberth A., Oliveira L.A.R., Siqueira Leite K.C., Freitas Bara M.T., Ghedini P.C., Bozinis M.C.V., Lobón G.S., Souza Gil E. (2018). Assessment of noni (*Morinda citrifolia* L.) product authenticity by solid state voltammetry. Int. J. Electrochem. Sci..

[10] Jin M.Y., Wang Y.X., Yang X.B., Yin H., Nie S.P., Wu X.Y. (2019). Structure characterization of a polysaccharide extracted from noni (*Morinda citrifolia* L.) and its protective effect against DSS-induced bowel disease in mice. Food Hydrocoll..

[11] Farias V.A., Lima A.D.R., Costa A.S., Freitas C.D.T., Araújo I.M.S., Garruti D.D.S., Figueiredo E.A.T., Oliveira H.D. (2020). Noni (*Morinda citrifolia* L.) fruit as a new source of milk-clotting cysteine proteases. Food Res. Int..

[12] Jamaludin R., Kim D.S., Salleh L.M., Lim S.B. (2021). Kinetic Study of Subcritical Water Extraction of Scopoletin, Alizarin, and Rutin from *Morinda citrifolia*. Foods.

[13] Kai K., Shimizu B., Mizutani M., Watanabe K., Sakata K. (2006). Accumulation of coumarins in Arabidopsis thaliana. Phytochemistry.

[14] Ahmed O.H., Hamad M.N., Jaafar N.S. (2017). Phytochemical investigation of Chenopodium murale (Family: *Chenopodiaceae*) cultivated in Iraq, isolation and identification of scopoletin and gallic acid. Asian J. Pharm. Clin. Res..

[15] Sethiya N.K., Trivedi A., Mishra S.H. (2015). Rapid validated high performance thin layer chromatography method for simultaneous estimation of mangiferin and scopoletin in *Canscora decussata* (South Indian *Shankhpushpi*) extract. Rev. Bras..

[16] Jamuna S., Karthika K., Paulsamy S., Thenmozhi K., Kathiravan S., Venkatesh R. (2015). Confertin and scopoletin from leaf and root extracts of *Hypochaeris radicata* have anti-inflammatory and antioxidant activities. Ind. Crops Prod..

[17] Ferdinal N., Alfajri R., Arifin B. (2015). Isolation and characterization of scopoletin from the bark of *Fagraea ceilanica* thumb and antioxidants tests. Int. J. Adv. Sci. Eng. Inf. Technol..

[18] Robe K., Izquierdo E., Vignols F., Rouached H., Dubos C. (2020). The Coumarins: Secondary Metabolites Playing a Primary Role in Plant Nutrition and Health. Trends Plant Sci..

[19] He B.T., Liu Z.H., Li B.Z., Yuan J.Y. (2022). Advances in biosynthesis of scopoletin. Microb. Cell Fact..

[20] Do C.T., Pollet B., Thévenin J., Silbout R., Denoue D., Barriere Y., Lapierre C., Jouanin L. (2007). Both caffeoyl coenzyme A 3-O-methyltransferase and caffeic acid O-methyltransferase 1 are involved in redundant functions for lignin, flavonoids, and sinapoyl malate biosynthesis in *A. thaliana*. Planta.

[21] Kai K., Mizutani M., Kawamura N., Yamamoto R., Tamai M., Yamaguchi H., Skata K., Shimizu B. (2008). Scopoletin is biosynthesized via ortho-hydroxylation of feruloyl CoA by a 2-oxolutarate-dependent dioxygenase in *Arabidopsis thaliana*. Plant J..

[22] Döll S., Kuhlmann M., Rutten T., Mette M.F., Scharfenberg S., Petridis A., Berreth D.C., Mock H.P. (2017). Accumulation of the coumarin scopolin under abiotic stress conditions is mediated by the *Arabidopsis thaliana* THO/TREX complex. Plant J..

[23] Zhao Y., Wang N., Sui Z., Huang C., Zeng Z., Kong L. (2019). The Molecular and Structural Basis of O-methylation Reaction in Coumarin Biosynthesis in *Peucedanum praeruptorum* Dunn. Int. J. Mol. Sci..

[24] Fellenberg C., Ohlen M., Handrick V., Vogt T. (2012). The role of CCoAOMT1 and COMT1 in Arabidopsis anthers. Planta.

[25] Liu H., Xu R.X., Zhang X.S., Zhu T.T., Lou H.X., Cheng A.X. (2019). The identification and functional characterization of three liverwort class I O-methyltransferases. Phytochemistry.

[26] Rafaliya R.V., Sakure A.A., Parekh M.J., Sushil K., Amarjeet S.S.T., Desai P.J., Patil G.B., Mistri J.G., Subhash N. (2021). Study of dynamics of genes involved in biosynthesis and accumulation of scopoletin at different growth stages of *Convolvulus prostratus* Forssk. Phytochemistry.

[27] Li H.M., Rotter D., Hartman T.G., Pak F.E., Havkin-Frenkel D., Belanger F.C. (2006). Evolution of novel O-methyltransferases from the *Vanilla planifolia* Caffeic Acid O-methyltransferase. Plant Mol. Biol..

[28] Yu X.M., Muhammad M.A., Li B.Q., Fang T., Chen F.X. (2021). Transcriptome data-based identification of candidate genes involved in metabolism and accumulation of soluble sugars during fruit development in ‘Huangguan’ plum. J. Food Biochem..

[29] Xu J.Y., Zhang Y., Qi D., Huo H.L., Dong X.G., Tian L.M., Liu C., Cao Y.F. (2021). Metabolomic and transcriptomic analyses highlight the influence of lipid changes on the post-harvest softening of *Pyrus ussurian* Max. ‘Zaoshu Shanli’. Genomics.

[30] Jia D.D., Lan Z.Q., Wu T. (2020). Ethylene is the key signal in the accumulation process of scopoletin in noni (*Morinda citrifolia*). Sci. Hortic..

[31] Rhoads A., Au K.F. (2015). PacBio Sequencing and Its Applications. Genom. Pro Teom. Bioinf..

[32] Li Y.P., Dai C., Hu C.G., Liu Z.C., Kang C.Y. (2017). Global identification of alternative splicing via comparative analysis of SMRT- and Illumina-based RNA-seq in strawberry. Plant J..

[33] Beiki H., Liu H., Huang J., Manchanda N., Nonneman D., Smith T.P.L., Reecy J.M., Tuggle C.K. (2019). Improved annotation of the domestic pig genome through integration of Iso-Seq and RNA-seq data. BMC Genom..

[34] Chao Q., Gao Z.F., Zhang D., Zhao B.G., Dong F.Q., Fu C.X., Liu L.J., Wang B.C. (2018). The developmental dynamics of the *Populus* stem transcriptome. Plant Biotechnol. J..

[35] Bart V.R., Lars R.K., Vincent R. (1998). Differential Cryptanalysis of the ICE Encryption Algorithm. Lect. Notes Comput. Sci..

[36] Salmela L., Rivals E. (2014). LoRDEC: Accurate and efficient long read error correction. Bioinformatics.

[37] Limin F., Niu B., Zhu Z.W., Wu S.T., Li W.Z. (2012). CD-HIT: Accelerated for clustering the next-generation sequencing data. Bioinformatics.

[38] Li W., Godzik A. (2006). Cd-hit: A fast program for clustering and comparing large sets of protein or nucleotide sequences. Bioinformatics.

[39] Xie C., Mao X.Z., Huang J.J., Ding Y., Wu J.M., Dong S., Kong L., Gao G., Li C.Y., Wei L.P. (2011). KOBAS 2.0: A web server for annotation and identification of enriched pathways and diseases. Nucleic Acids Res..

[40] Huma N., Veda P.P., Swati S., Upendra N.D. (2013). Structure-function analyses and molecular modeling of caffeic acid-O-methyltransferase and caffeoyl-CoA-O-methyltransferase: Revisiting the basis of alternate methylation pathways during monolignol biosynthesis. Biotechnol. Appl. Bioc..

[41] Ye Z.H., Zhong R.Q., Herbert Morrison W., Himmelsbach D.S. (2001). Caffeoyl coenzyme A O-methyltransferase and lignin biosynthesis. Phytochemistry.

[42] Gatadi S., Gour J., Nanduri S. (2019). Natural product derived promising anti-MRSA drug leads: A review. Bioorg. Med. Chem..

[43] Kapoor L., Simkin A.J., Doss G.P., Siva R. (2022). Fruit ripening: Dynamics and integrated analysis of carotenoids and anthocyanins. BMC Plant Biol..

[44] Julie C., Rachel B., Corinne S., Roland B., Bernard F., Patrick S. (2002). Down regulation of a Pathogen-Responsive Tobacco UDP-Glc:Phenylpropanoid Glucosyltransferase Reduces Scopoletin Glucoside Accumulation, Enhances Oxidative Stress, and Weakens Virus Resistance. Plant Cell.

[45] Vivek Y., Wang Z.Y., Wei C.H., Aduragbemi A., Bilal A., Yang X.Z., Zhang X. (2020). Phenylpropanoid Pathway Engineering: An Emerging Approach towards Plant Defense. Pathogens.

[46] Wu C., Zuo D., Xiao S., Wang Q., Cheng H., Lv L., Zhang Y., Li P., Song G. (2021). Genome-Wide Identification and Characterization of GhCOMT Gene Family during Fiber Development and Verticillium Wilt Resistance in Cotton. Plants.

[47] Seong E.S., Yoo J.H., Lee J.G., Kim H.Y., Hwang I.S., Heo K., Kim J.K., Lim J.D., Sacks E.J., Yu C.Y. (2013). Antisense-overexpression of the MsCOMT gene induces changes in lignin and total phenol contents in transgenic tobacco plants. Mol. Biol. Rep..

[48] Rubio-Covarrubias O.A., Douches D.S., Hammerschmidt R., daRocha A., Kirk W.W. (2006). Effect of photoperiod and temperature on resistance against *Phytophthora infestans* in susceptible and resistant potato cultivars: Effect on deposition of structural phenolics on the cell wall and resistance to penetration. Am. J. Potato Res..

[49] Abdelali B., Alex C., Norzawani B.M.Y., Joseph S.P., Sun Z.C., John E.C. (2011). Comparative genomics and evolutionary analyses of the O-methyltransferase gene family in Populus. Gene.

[50] Liu Y.S., Wang Y.Z., Pei J.B., Li Y.D., Sun H.Y. (2021). Genome-wide identification and characterization of COMT gene family during the development of blueberry fruit. BMC Plant Biol..

[51] Paul D., Christopher M., Marta M., Helena O., Catherine L., Robbie W., Jennifer S., David M., Abdellah B., Yukiko T. (2019). RNAi-suppression of barley caffeic acid O-methyltransferase modifies lignin despite redundancy in the gene family. Plant Biotechnol. J..

[52] Hawkins S., Boudet A., Grima-Pettenati J. (2003). Characterisation of caffeic acid O-methyltransferase and cinnamyl alcohol dehydrogenase gene expression patterns by in situ hybridisation in *Eucalyptus gunnii* Hook. Plantlets. Plant Sci..

[53] Hamberger B., Ellis M., Friedmann M., Souza C.A., Barbazuk B., Douglas C.J. (2007). Genome-wide analyses of phenylpropanoid-related genes in *Populus trichocarpa*, *Arabidopsis thaliana*, and *Oryza sativa*: The Populus lignin toolbox and conservation and diversification of angiosperm gene families. Can. J. Bot..

[54] Xu R.X., Zhao Y., Gao S., Zhang Y.Y., Li D.D., Lou H.X., Cheng A.X. (2015). Functional characterization of a plastidal cation-dependent O-methyltransferase from the liver-wort Plagiochasma appendiculatum. Phytochemistry.

[55] Widiez T., Hartman T.G., Dudai N., Yan Q., Lawton M., Havkin-Frenkel D., Belanger F.C. (2011). Functional characterization of two new members of the caffeoyl CoA O-methyltransferase-like gene family from Vanilla planifolia reveals a new class of plastid-localized O-methyltransferases. Plant Mol. Biol..

[56] Li L.L., Tao S.T., Zhang H.W., Huang W.J., Dunwell J.M., Li M. (2021). Identification and Characterization of the CCoAOMT Gene Family in Apple, Chinese White Pear, and Peach. J. Am. Soc. Hortic. Sci..

[57] Liu Y.X., Zou D.M., Wu B.S., Lin D.H., Zhang Z.H., Wu J.C. (2015). Cloning and expression analysis of a CCoAOMT homolog in loquat fruit in response to low-temperature storage. Postharvest Biol. Technol..

[58] Zhang X.S., Ni R., Wang P.Y., Zhu T.T., Sun C.J., Lou H.X., Cheng A.X. (2019). Isolation and functional characterization of two Caffeoyl Coenzyme A 3-O- methyltransferases from the fern species *Polypodiodes amoena*. Plant Physiol. Bioch..

[59] Yao R.L., Zhao Y.C., Liu T.T., Huang C.L., Xu S., Sui Z.W., Luo J., Kong L.Y. (2017). Identification and functional characterization of a p-coumaroyl CoA 2’-hydroxylase involved in the biosynthesis of coumarin skeleton from Peucedanum praeruptorum Dunn. Plant Mol. Biol..

[60] Jia D.D., Lan Z.Q., Wu T. (2018). Studies on genetic transformation of seedlessness gene to noni (*Morinda citrifolia*) with its stem segments as the expalnts. J. Southwest..

[61] Liu J., Lan Z.Q., Wu T., Yang Z.Y. (2018). Transformation of non-seeded gene into noni root segment by Agrobacterium. Biotechnology.

