# Supplementary material for: RNA-Seq and Iso-Seq Reveal the Important Role of COMT and CCoAOMT Genes in Accumulation of Scopoletin in Noni (Morinda citrifolia)
Source: Genes (Basel). 2022 Oct 31;13(11):1993. doi: 10.3390/genes13111993 (PMC9689816; doi:10.3390/genes13111993)
Supplement: Supplementary file 1 [file genes-13-01993-s001.zip › supplement figures.pdf]

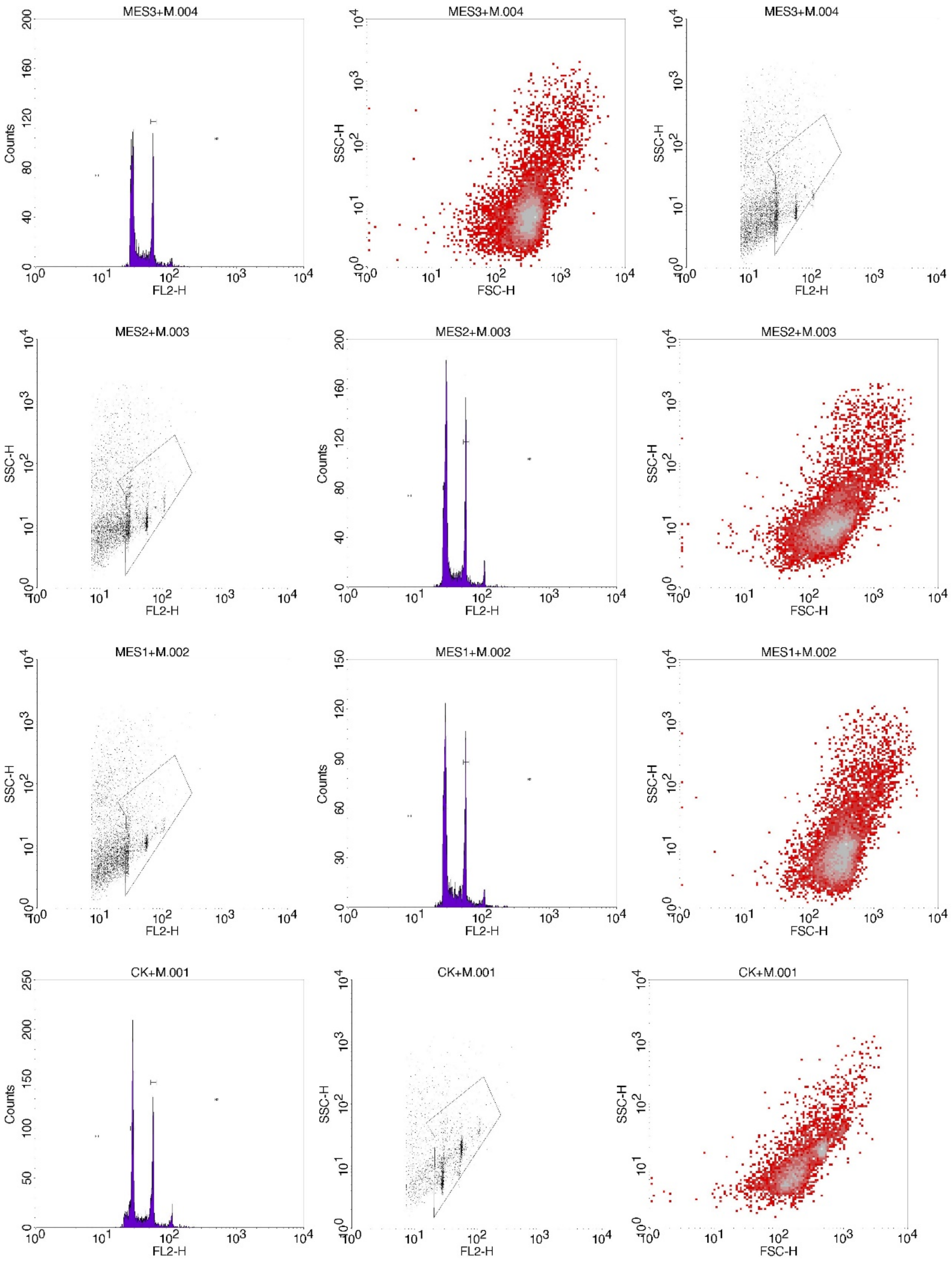

Figure S1 Results of genome size determination.

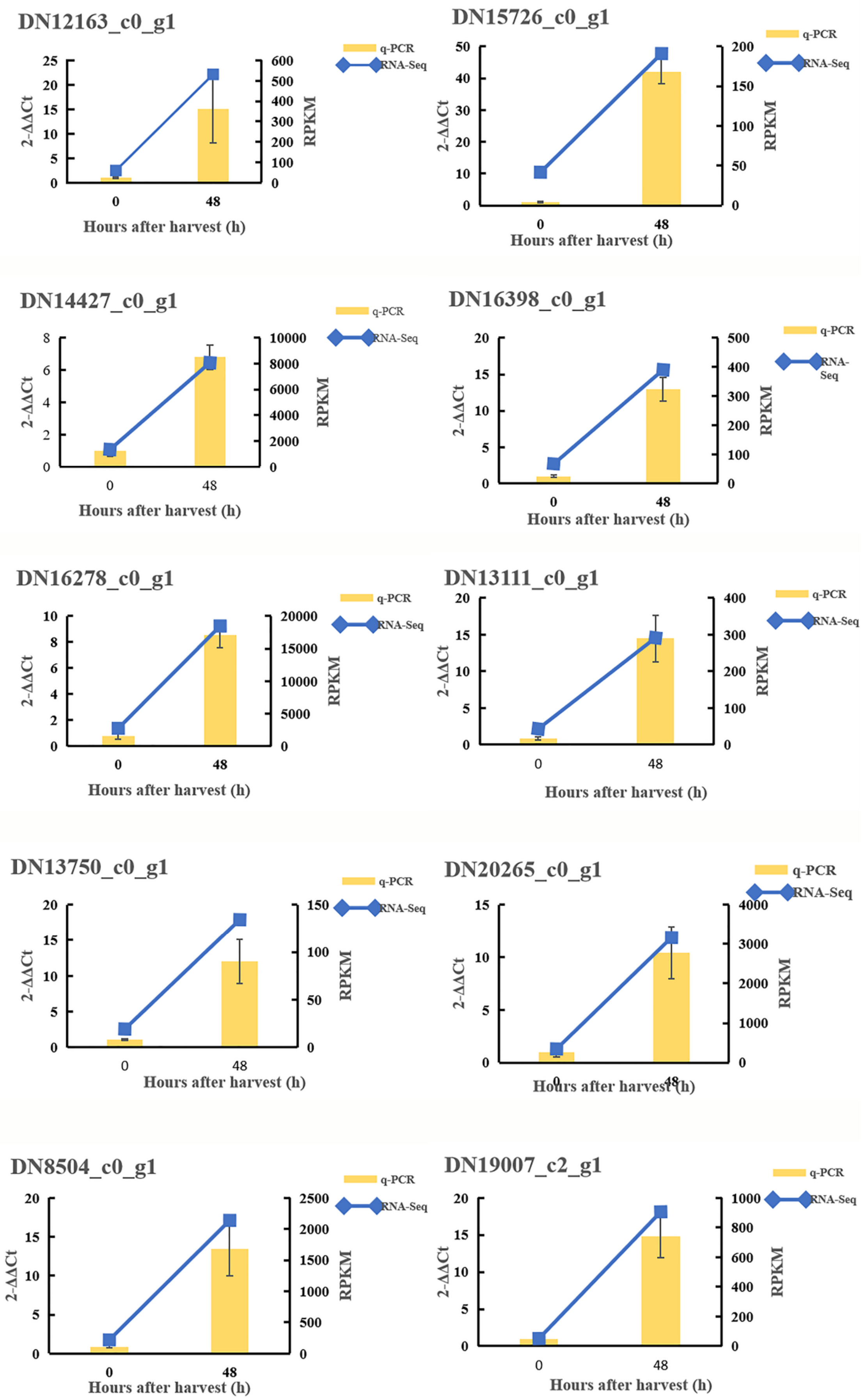
DN12163_c0_g1
DN15726_c0_g1
DN14427_c0_g1
DN16398_c0_g1
DN16278_c0_g1
DN13111_c0_g1
DN13750_c0_g1
DN20265_c0_g1
DN8504_c0_g1
DN19007_c2_g1
q-PCR
RNA-Seq
2-ΔΔCt
RPKM
0
48
Hours after harvest (h)

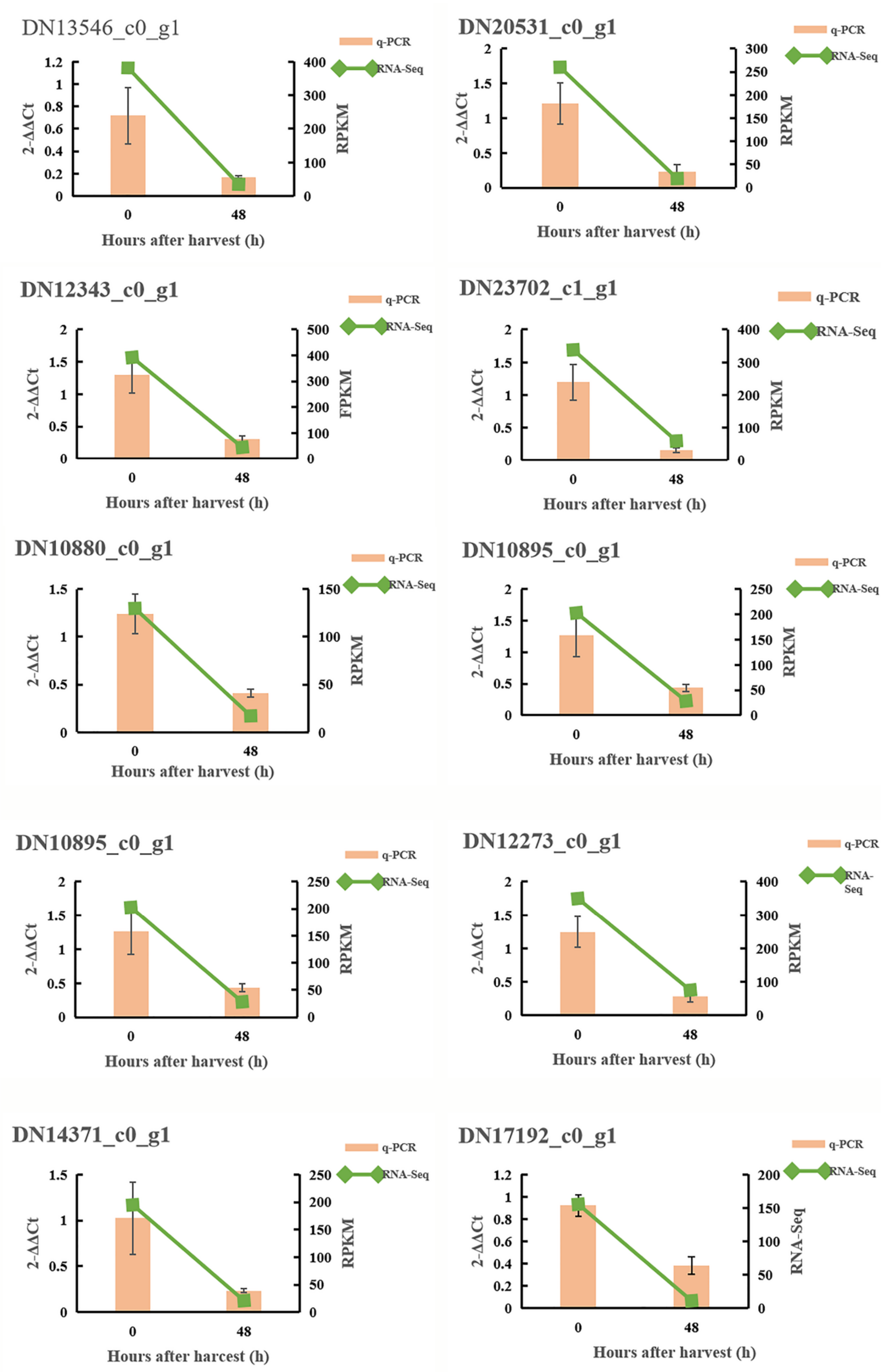

Figure S2 q-PCR verification of RNA-Seq (10 up regulated and 10 down regulated genes).
